# Mitochondrial peptides modulate mitochondrial function during cellular senescence

**DOI:** 10.18632/aging.101463

**Published:** 2018-06-10

**Authors:** Su-Jeong Kim, Hemal H. Mehta, Junxiang Wan, Chisaka Kuehnemann, Jingcheng Chen, Ji-Fan Hu, Andrew R. Hoffman, Pinchas Cohen

**Affiliations:** 1 Leonard Davis School of Gerontology, University of Southern California, Los Angeles, CA 90089, USA; 2 Buck Institute for Research on Aging, Novato, CA 94945, USA; 3 Stanford University Medical School, Palo Alto Veterans Institute for Research, Palo Alto, CA 94304, USA

**Keywords:** mitochondria, senescence, mitochondrial-derived peptides, mitochondrial energetics, mtDNA methylation, SASP (senescence-associated secretory phenotype)

## Abstract

Cellular senescence is a complex cell fate response that is thought to underlie several age-related pathologies. Despite a loss of proliferative potential, senescent cells are metabolically active and produce energy-consuming effectors, including senescence-associated secretory phenotypes (SASPs). Mitochondria play crucial roles in energy production and cellular signaling, but the key features of mitochondrial physiology and particularly of mitochondria-derived peptides (MDPs), remain underexplored in senescence responses. Here, we used primary human fibroblasts made senescent by replicative exhaustion, doxorubicin or hydrogen peroxide treatment, and examined the number of mitochondria and the levels of mitochondrial respiration, mitochondrial DNA methylation and the mitochondria-encoded peptides humanin, MOTS-c, SHLP2 and SHLP6. Senescent cells showed increased numbers of mitochondria and higher levels of mitochondrial respiration, variable changes in mitochondrial DNA methylation, and elevated levels of humanin and MOTS-c. Humanin and MOTS-c administration modestly increased mitochondrial respiration and selected components of the SASP in doxorubicin-induced senescent cells partially via JAK pathway. Targeting metabolism in senescence cells is an important strategy to reduce SASP production for eliminating the deleterious effects of senescence. These results provide insight into the role of MDPs in mitochondrial energetics and the production of SASP components by senescent cells.

## Introduction

Mitochondria play important roles in cellular energy production, metabolism, and cellular signaling. These organelles have their own genomes that contain 13 mRNAs, 22 tRNAs, and 2 rRNAs to produce proteins that participate in oxidative phosphorylation. Epigenetic modification of mitochondrial DNA, including DNA methylation, is still controversial. The overall mitochondrial DNA methylation occurs at a lower frequency compared to nuclear DNA, but specific locations have been found to be differentially methylated in certain cellular conditions or in different biological samples [1,2]. For example, the ND6 and 12S rRNA region showed the increased levels of methylation following oxidative stress and environmental pollution, respectively [2,3]. Higher methylation of a region typically suppresses the expression of adjacent genes. DNA methyltransferase 1 (DNMT1) is localized in the mitochondria, and the expression of DNMT1 alters during the oxidative stress [3,4]. During aging, the methylation of the 12S rRNA region, but not 16S rRNA region, decreased with age [2,5]. Emerging studies show that mitochondrial DNA (mtDNA) also has small open reading frames encoding mitochondrial-derived peptides (MDPs) including humanin, MOTS-c, and SHLP1-6 [6–8]. MDPs are detected in multiple tissues and plasma, and they regulate mitochondrial bioenergetics and mitochondrial metabolism [8,9]. They play cytoprotective roles in age-related diseases, including cardiovascular diseases, diabetes and Alzheimer’s disease [10].

Humanin is a 24-amino acid peptide encoded within the 16S rRNA region of the mtDNA. It is secreted in response to cellular stress and has broad cytoprotective and neuroprotective effects in various diseases such as atherosclerosis, Alzheimer's disease and type 2 diabetes [9–14]. Cell surface receptors have been identified for humanin that activate signaling pathways, including JAK/STAT, ERK1/2 and RAC-alpha serine/threonine-protein kinase (AKT), which are associated with cell proliferation and survival [15–17]. Humanin blocks apoptosis, decreases inflammation, and reduces oxidative stress in various aging models [18–22].

MOTS-c is a 16-amino acid peptide encoded within the 12S rRNA region of mtDNA. MOTS-c dramatically increases endogenous 5-aminoimidazole-4-carboxamide ribonucleotide (AICAR) levels and activates 5' AMP-activated protein kinase (AMPK) [8]. MOTS-c increases glucose utilization, fatty acid oxidation, and alters mitochondrial function and nucleotide metabolism [8]. MOTS-c suppresses ovariectomy-induced osteoporosis via AMPK activation [8,23]. MOTS-c improves metabolic functions and have shown as potential biomarkers for metabolic function. MOTS-c decreases fat accumulation in the liver in high-fat diet-induced obese mice. MOTS-c levels are inversely correlated with insulin sensitivity in lean, not obese, individuals. Another study showed that circulating MOTS-c levels are reduced in obese male children and adolescents, but not in obese female. MOTS-c levels are inversely correlated with markers of insulin resistance and obesity.

Among the basic processes that are known to drive aging phenotypes and pathology are genomic instability, epigenetic alterations, mitochondrial dysfunction and cellular senescence [24]. Although humanin and MOTS-c have protective roles in multiple age-associated diseases, the roles of these peptides in cellular senescence have not been explored.

Cellular senescence plays an important role in wound healing and cancer suppression, as well as contributing to age-related tissue dysfunction [25,26]. Senescent cells accumulate with age in most, if not all, vertebrate tissues, and are thought to promote many diseases of aging [27]. Senescent cells show an irreversible cell cycle arrest and adopt senescence-associated secretory phenotypes (SASPs), which include pro-inflammatory cytokines, chemokines, proteases and growth factors. These factors can maintain the cell cycle arrest in an autocrine manner, and induce senescence in neighboring cells in a paracrine manner [28,29]. When senescent cells are not removed by the immune system, the SASP can cause chronic inflammation, alter tissue structure and function and increase the risk of cancer [30,31]. This chronic inflammation is associated with mortality and multiple age-associated diseases [32]. The identification of a new class of drugs, senolytics, which selectively kill senescent cells or modulate the SASP, will soon enter clinical trial. Indeed, the elimination of senescent cells in transgenic mice delays age-related pathologies, including hepatic steatosis, cataract formation, skin thinning and age-related fat loss, and increases median life span in mouse models [33–37]. Some senolytics induce apoptosis of senescent cells by inhibiting anti-apoptotic proteins, including BCL-2 and Bcl-XL or preventing FOXO4 from binding to p53 [38–40]. Since interest in pharmacological and genetic manipulations to induce apoptosis in senescent cells is growing, it will be important to identify novel senolytics and validate their mechanisms and targets.

Senescent cells are metabolically active, producing energy-consuming effectors of senescence, despite the loss of proliferative activity [41]. Depending on the inducer, senescent cells show higher levels of glycolysis [42], fatty acid oxidation and mitochondrial respiration [43]. Manipulating bioenergetic status can induce senescence and a SASP, suggesting that bioenergetics play a role in the senescence phenotype [41,44,45]. Thus, altering the metabolic status of senescence cells may be an important strategy for eliminating the deleterious effects of senescence. Indeed, metabolism-targeting drugs (e.g., rapamycin and etomoxir) have been used to eliminate senescent tumor cells following chemotherapy, leading to suppressed SASP production [43,46,47]. Humanin and MOTS-c impact cellular energetics. Humanin increases mitochondrial biogenesis and respiration in human retinal pigment epithelial cells [20]. MOTS-c increases glycolysis and decreases mitochondrial respiration in HEK293 cells [8]. As humanin and MOTS-c alleviate age-associated pathologies and impact mitochondrial energetics, we hypothesized that these mitochondrial-derived peptides influence senescence phenotypes in energy-demanding senescent cells.

In this study, we investigate mitochondrial energetics, mtDNA methylation and MDP levels in senescent cells, and evaluate the potential of humanin and MOTS-c as novel senolytics or SASP modulators that can alleviate symptoms of frailty and extend health span by targeting mitochondrial bioenergetics.

## RESULTS

### Mitochondrial biogenesis and energetics are altered during doxorubicin-induced senescence

**Figure 1:**
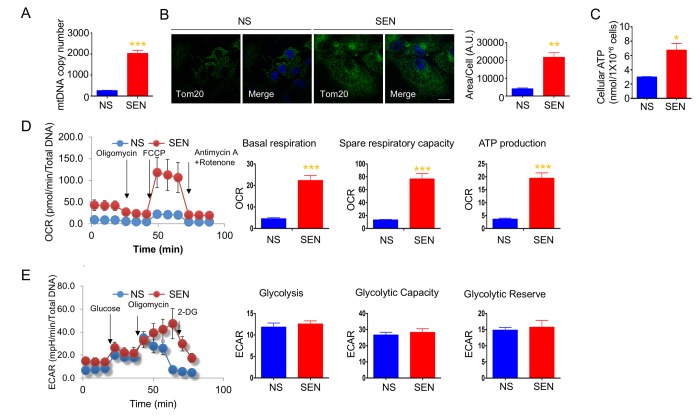
**Mitochondria mass and energetics are altered during doxorubicin-induced senescence.** (**A**) mitochondrial DNA (mtDNA) copy number in non-senescent (quiescent) and senescent cells. (**B**) Representative images of Tom20 (green; mitochondria) and Hoechst 33258 (blue; nucleus) immunostaining in non-senescent (quiescent) and senescent cells. Scale bar, 20 μm. The area of Tom20 staining per cells were measured using image J. (**C**) Cellular ATP levels in non-senescent (quiescent) and senescent cells. (**D**) Cellular oxygen consumption rate (OCR) in non-senescent and senescent cells. The basal respiration, spare respiratory capacity, and ATP production are calculated based on the sequential compound injection according to the manufacture’s instruction. (**E**) The extracellular acidification rate (ECAR) in non-senescent (quiescent) and senescent cells. Glycolysis, glycolytic capacity, and glycolytic reserve are calculated based on the sequential compound injection according to the manufacture’s instruction. Data are reported as mean ± SEM of three to eight independent experiments. Significant differences were determined by Student’s *t*-tests. *p<0.05, ***p<0.001. Abbreviations: NS, Non-senescent cells (quiescent); SEN, Senescent cells.

To understand changes in mitochondrial biogenesis and energetics during cellular senescence, we investigated mitochondrial number, ATP production, and oxidative respiration in cellular senescence models. We used doxorubicin- and hydrogen peroxide-induced senescence of human primary adult dermal fibroblasts (HDFa) as DNA damage response models. The quiescent cells were used as a control for the mitochondrial biogenesis and energetics studies. Doxorubicin-induced senescent cells had higher mitochondria copy numbers compared to non-senescent cells (Fig. 1A). Immunofluorescence of mitochondria using mitochondrial outer membrane protein, mitochondrial import receptor subunit TOM20 antibodies showed that the area of TOM20 staining is more, and mitochondria appeared to be connected in the senescent cells (Fig. 1B). To determine whether the increased mitochondria mass increases energy production in senescence cells, we measured cellular ATP levels, and observed that senescent cells produced more ATP compared to non-senescent cells (Fig. 1C). To understand whether higher ATP production was due to mitochondrial respiration from more mitochondria or increased glycolysis, we measured the real-time oxygen consumption rate (OCR) and extracellular acidification rate (ECAR). Senescent cells showed higher basal OCR, spare respiratory capacity, and greater ATP production compared to non-senescent proliferating cells (Fig. 1D). We also examined a different cell line and model of senescence. Hydrogen peroxide-induced senescence in primary dermal fibroblasts (HDFa) also demonstrated higher basal OCR (Fig. S1). Moreover, doxorubicin-induced senescence in HCA2 (primary foreskin) fibroblasts demonstrated increased basal OCR: non-senescent cells consumed 37.77 ± 2.94 pmoles/min; senescent cells consumed 63.3 ± 8.22 pmoles/min. These results demonstrate that elevated mitochondrial respiration may occur in senescent cells. Glycolysis, glycolytic capacity and glycolytic reserve were not altered in senescent cells. However, there was a trend for ECAR to increase in senescent cells (Fig. 1E). Both spare respiratory capacity and glycolytic capacity seem to be increased in senescent cells, suggesting that senescent cells are more resistant to metabolic challenges. These results suggest that mitochondrial number and energy production play important roles in energy production to support the energy demands of senescent cells.

### Mitochondria depend more on glucose and fatty acid utilization during doxorubicin-induced cellular senescence

**Figure 2:**
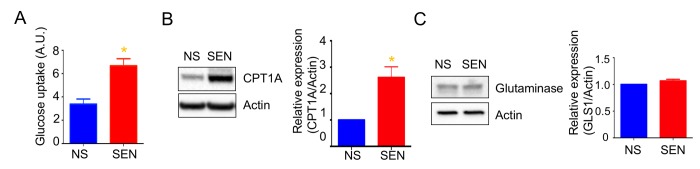
**Mitochondrial fuel usage is altered during doxorubicin-induced senescence.** (**A**) Glucose uptake rate was measured by 2-NBDG, a fluorescently labeled deoxyglucose analog. Quantification and representative western blots of (**B**) carnitine palmitoyltransferase I (CPT1A) and (**C**) glutaminase (GLS1), both showing beta-actin as a loading control. Data are reported as mean ± SEM of three to six independent experiments. Significant differences were determined by Student’s *t*-tests. *p<0.05. Abbreviations: NS, Non-senescent cells (quiescent); SEN, Senescent cells.

Mitochondria use three different fuels for energy production: glucose, glutamine, and fatty acids. We therefore determined fuel utilization and mitochondrial respiration in senescent cells. Senescent cells showed a trend toward increased glucose and fatty acid utilization and decreased glutamine utilization (Fig. S2). Further, glucose uptake increased in senescent cells (Fig. 2A). These results suggest that the glucose utilized more in the mitochondrial respiration. In agreement with higher fatty acid utilization, the expression of carnitine palmitoyltransferase I (CPT1), which facilitates translocation of long chain fatty acids into mitochondria for beta oxidation, increased in senescent cells compared to non-senescent cells (Fig. 2B). We also examined protein levels of glutaminase (GLS1), which converts glutamine to glutamate for subsequent conversion to alpha-ketoglutarate and oxidation by the TCA cycle. GLS1 protein levels remained unaltered in senescent cells (Fig. 2C).

### Glycolysis but not mitochondrial respiration increased during replicative senescence

**Figure 3:**
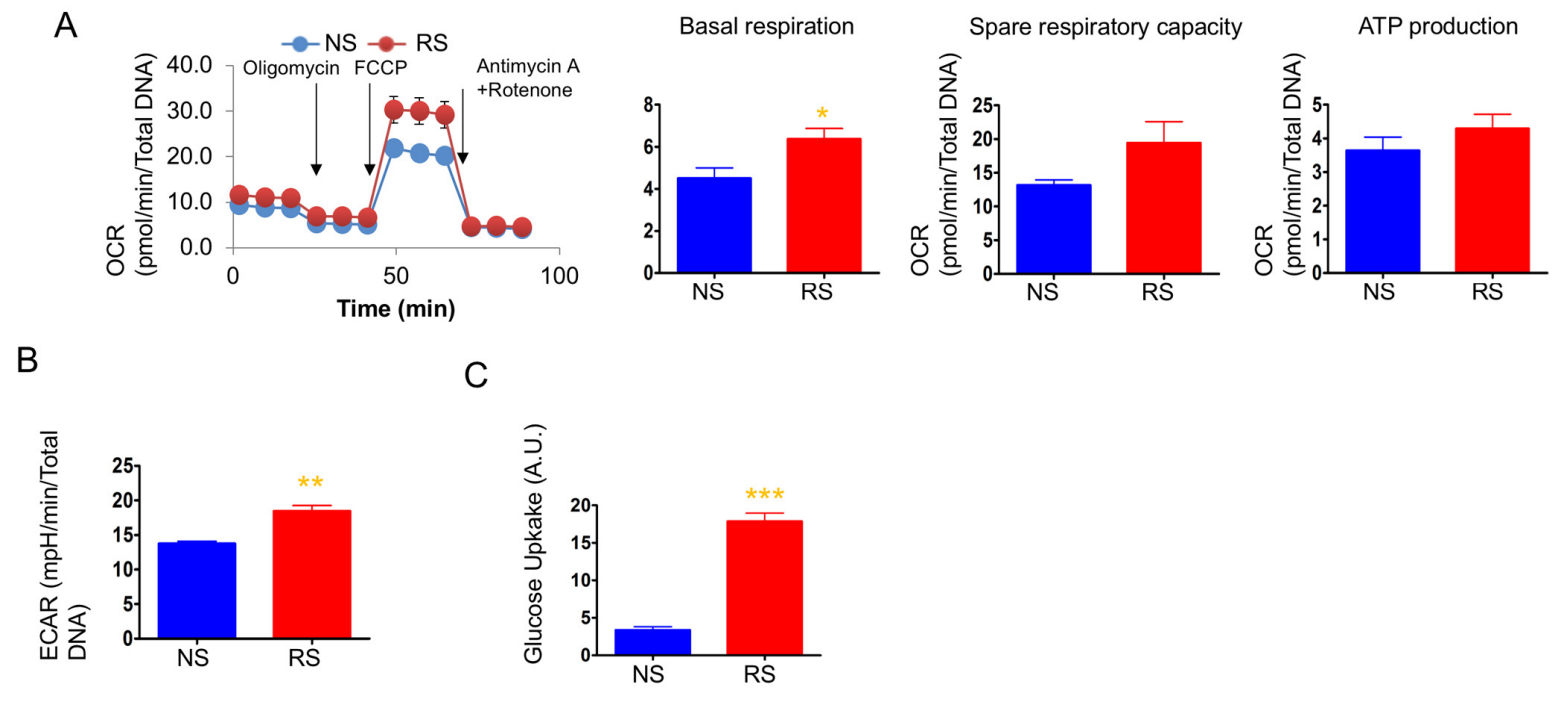
**Mitochondrial respiration was not altered, but glycolysis was enhanced in replicative senescence.** (**A**) Cellular oxygen consumption rate (OCR) in non-senescence (quiescent) and senescent cells. The basal respiration, spare respiratory capacity, and ATP production are calculated based on the sequential compound injection according to the manufacture’s instruction. (**B**) The extracellular acidification rate (ECAR) in non-senescent (quiescent) and senescent cells. (**C**) Glucose uptake rate were measured by 2-NBDG, a fluorescently labeled deoxyglucose analog. Data are reported as mean ± SEM of three to eight independent experiments. Significant differences were determined with Student’s *t*-tests. *p<0.05, **p<0.01, ***p<0.001. Abbreviations: NS, Non-senescent cells (quiescent); SEN, Senescent cells.

We next measured OCR and ECAR to determine the cellular energetics of replicatively senescent primary human dermal fibroblasts. As opposed to doxorubicin-induced senescent cells, replicatively senescent cells showed levels of mitochondrial respiration similar to non-senescent cells, although basal respiration was slightly higher than in non-senescent cells (Fig. 3A). Consistent with other studies, replicatively senescent primary dermal fibroblasts showed increased glycolysis and, expectedly, dramatically increased glucose uptake (Figs. 3B and 3C). Senescent cells have high energy demand to increase the size of the cells and produce SASPs. Replicatively senescent cells have been shown to utilize glycolysis to supply the energy for those cellular processes [42,48,49]. On the other hand, doxorubicin-induced senescent cells show an increase in mitochondrial mass and utilized mitochondrial respiration. During DNA damage-induced senescence, mitochondria play important roles not only for respiration and ROS production, but also for DNA damage response signaling [50]. Thus, the expansion of the mitochondria and mitochondrial function are vital for doxorubicin-induced senescent phenotypes. The increased mitochondrial mass and enhanced mitochondrial function found in DNA damage signaling-mediated senescent such as doxorubicin, may not occur in replicatively senescent cells and, therefore, the enhanced glycolysis may be the main energy supply for replicatively senescent cells.

Several studies showed that doxorubicin per se alters mitochondrial respiration and glycolysis. Doxorubicin increased several glycolytic enzymes and increased glycolytic activity, and decreased TCA cycle and fatty acid oxidation in rat heart after 6 weekly injections of doxorubicin [51,52]. Doxorubicin increased the mRNA of TCA cycle relevant enzymes in rat perfused heart [53]. Although the effects of doxorubicin on TCA cycle are contradictory, the increased mitochondrial respiration in doxorubicin-induced senescent cells can be driven by the gene expression by doxorubicin as well as the increase of mitochondrial mass.

### Mitochondrial DNA methylation regulates mitochondrial-encoded genes expression

**Figure 4:**
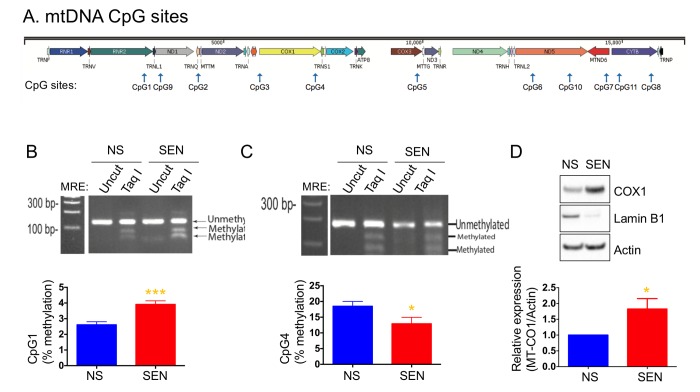
**Mitochondria DNA methylation changes during doxorubicin-induced senescence.** (**A**) Schematic diagram of mitochondrial genes and CpG sites in the mtDNA. Quantification and representative agarose gel images of mtDNA methylation levels at the site of (**B**) CpG1 and (**C**) CpG4. (**D**) Quantification and representative western blots of COX1 (MT-CO1) in non-senescent and senescent cells. Reduced lamin B1 was used as a senescence marker. Data are reported as mean ± SEM of three to four independent experiments. Significant differences were determined by Student’s *t*-tests. *p<0.05, ***p<0.001. Abbreviations: NS, Non-senescent cells (quiescent); SEN, Senescent cells.

Mammalian mitochondrial DNA is methylated at CpG sites, although the importance of this phenomenon is unknown (Fig. 4A). We recently reported that several mtDNA CpG sites were methylated during senescence [54]. Here, we examined the methylation status of CpG sites 1, 2 and 4 in doxorubicin-induced senescence. CpG1 showed a higher level of methylation, whereas CpG4 showed a lower level of methylation in senescent cells compared to non-senescent cells (Figs. 4B and 4C). The methylation level at the CpG2 site was not changed (*not shown*). Increased DNA methylation typically correlates with decreased gene expression. However, methylation of CpG4, which is present in the MT-CO1 gene, decreased, and COX1 expression increased in senescent cells (Fig. 4D). The increased level of COX1 is consistent with increased mitochondria mass and mitochondrial respiration in senescent cells.

### Mitochondrial-derived peptides are altered during senescence

**Figure 5:**
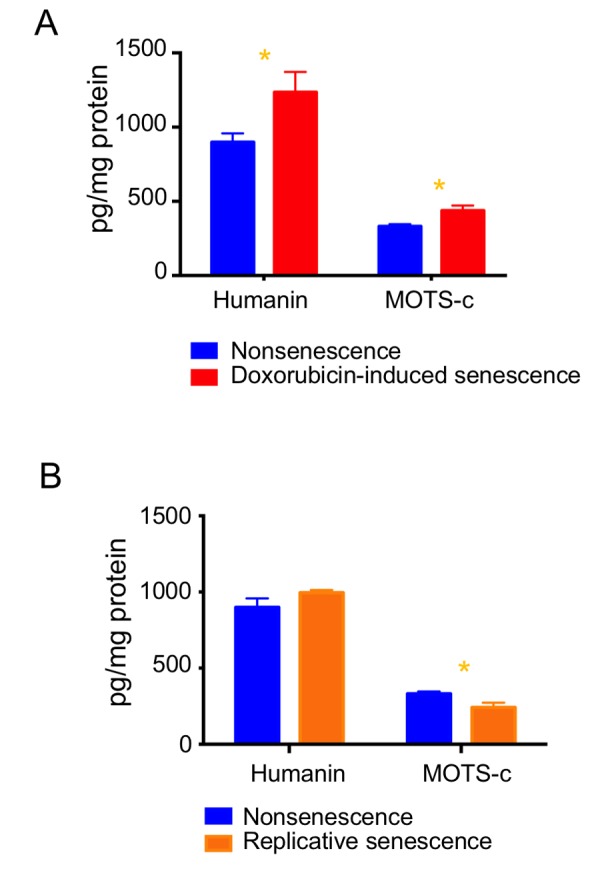
**Expression levels of MDPs are differentially regulated during cellular senescence.** Humanin and MOTS-c levels were examined in (**A**) doxorubicin-induced senescence and (**B**) replicative senescence. Data are reported as mean ± SEM of three independent experiments. Significant differences were determined by Student’s *t*-tests. *p<0.05.

To determine whether MDP levels varied during senescence, we measured humanin, SHLP2 and SHLP6, which are derived from the 16S rRNA region, and MOTS-c, which is derived from the 12S rRNA region, by ELISA. The levels of humanin and MOTS-c increased (Fig. 5A), but SHLP2 and SHLP6 levels remained unchanged during doxorubicin-induced senescence (Figs. S3A-S3B). Humanin levels were not changed in replicative senescence, whereas MOTS-c levels slightly decreased (Fig. 5B). As these four MDPs were differentially regulated, we speculate that the increased mitochondria mass itself is not responsible for elevating humanin and MOTS-c levels in doxorubicin-induced senescence.

### Humanin and MOTS-c regulate SASP production

Humanin and MOTS-c administration showed beneficial effects on age-related diseases in mice [10,55]. We hypothesized that humanin and MOTS-c can kill senescent cells and tested the potential role of these peptides as senolytics. To understand the effect of humanin and MOTS-c in doxorubicin-induced senescent cells, we examined cell death, mitochondrial respiration and SASP production following treatment with these peptides. We treated quiescent and doxorubicin-induced senescent cells with 1 and 10μM humanin and MOTS-c peptides for 3 days and examined the number of cells and cell death. Neither peptide altered the number of cells after treatment (*data not shown*). In addition, humanin did not induce caspase-3 dependent apoptosis in senescent cells (Fig. S4A), and MOTS-c treatment had no effect on caspase-3 dependent apoptosis in non-senescent and doxorubicin-induced senescent cells (*data not shown*). We used Annexin V and PI staining and MTT assay to examine the cell death following humanin treatment. We did not see the cell death by humanin in both doxorubicin-induced and replicatively senescent cells (Figs. S4B-4C).

Humanin and MOTS-c can modulate mitochondrial energetics. Since MOTS-c reduced mitochondrial respiration in HEK293 cells, we hypothesized that MOTS-c would decrease the elevated mitochondrial respiration in senescent cells and reduce the SASP production.

**Figure 6:**
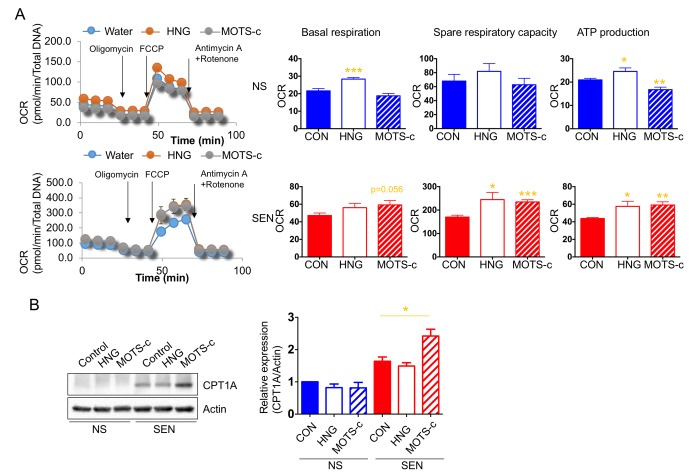
**Humanin and MOTS-c modulate the mitochondrial respiration.** (**A**) Cellular oxygen consumption rate (OCR; pmole/min/total DNA) in non-senescence (quiescent) and senescent cells in the absence or presence of either HNG (a potent analogue of humanin with a glycine substitution, S14G) or MOTS-c. The basal respiration, spare respiratory capacity, and ATP production are calculated based on the sequential compound injection according to the manufacture’s instruction. (**B**) Representative western blots of carnitine palmitoyltransferase I (CPT1A) in non-senescence and senescent cells in the absence or presence of either HNG (a potent analogue of humanin with a glycine substitution, S14G) or MOTS-c. Quantification of carnitine palmitoyltransferase I (CPT1A) expression. Data are reported as mean ± SEM of three independent experiments. Significant differences were determined with one-way ANOVA followed by Tukey’s *post hoc* test. *p<0.05.

In non-senescent cells, humanin increases whereas MOTS-c decreases mitochondrial respiration. Unexpectedly, humanin and MOTS-c both increased mitochondrial respiration in senescent cells (Fig. 6A). Additionally, we showed that senescent cells depend more on glucose and fatty acid utilization to support the elevated mitochondrial respiration. Because humanin and MOTS-c treatment increased mitochondrial respiration in senescent cells, we examined whether these peptides increase fatty acid utilization in these cells. Specifically, we examined the level of CPT1 in the presence or absence of these peptides.

MOTS-c, but not humanin, increased CPT1 levels in senescent, but not non-senescent, cells (Fig. 6B). This finding suggests that MOTS-c increases fatty acid utilization in senescent cells to further increase mitochondrial respiration.

Next, we examined the levels of a small subset of SASP factors in non-senescent (quiescent) and senescent cells following humanin and MOTS-c treatment. Exposure to these peptides increased production of the SASP factors IL-6, IL-1β, IL-8, IL-10 and TNFα (Table 1). We also examined the effects of humanin and MOTS-c in replicatively senescent cells. Humanin and MOTS-c treatment increased the level of these SASP factors in replicatively senescent cells (Table 2). Because trifluoroacetic acid (TFA) is a common contaminant in purification of peptide, trace amounts of TFA may affect our assays. We examined whether TFA alters the SASP of senescent cells and found that 40μM TFA did not alter SASP production such as IL-6, IL-1β, IL-8, IL-10 and TNFα (*data not shown*), indicating that humanin and MOTS-c increase SASP factors independent of TFA. Humanin activates JAK/STAT pathways through gp130 receptor complexes [15]. We treated cells with humanin in the presence or absence of JAK inhibitor to understand whether JAK pathways are involved in SASP production by humanin. JAK inhibitor treatment diminished the IL-6 production induced by humanin (Table 3). However, IL-10 and TNF-α levels were not altered by the JAK inhibitor. These results suggest that other signaling pathways may be involved in SASP production, and deciphering these signaling pathways will be informative. Humanin and MOTS-c increased a subset of SASP factors in both non-senescent and senescent cells, which raises the question of whether humanin and MOTS-c can increase cytokine production in general. To understand whether these peptides specifically upregulate SASP, we measured cytokines which have not been demonstrated to be induced during cellular senescence including eotaxin, eotaxin-3, IFN-γ, and I-TAC, in doxorubicin-induced and replicatively senescent dermal fibroblast cells. The levels of eotaxin, IFN-γ, and I-TAC were too low to be detected in the non-senescent and senescent cells, and eotaxin-3 levels were not altered by humanin and MOTS-c (table 4). These results suggest that humanin and MOTS-c specifically increases a small subset of SASP factors during senescence.

**Table 1:** Cytokine levels in non-senescent and doxorubicin-induced senescent cells.

		Water		HNG		MOTS-c	
		Mean	SE	Mean	SE	Mean	SE
IL-6	NS	0.46	0.06	0.78	0.11	1.98^***^	0.22
	SEN	1.51	0.26	3.37^**^	0.32	6.40^***^	0.38
IL-1beta	NS	0.10	0.03	0.35^*^	0.09	1.05^***^	0.03
	SEN	1.17	0.10	2.21	0.44	3.56^*^	0.82
IL-8	NS	0.08	0.02	0.20^**^	0.01	0.83^***^	0.09
	SEN	1.10	0.17	1.60	0.30	2.20	0.79
IL-10	NS	0.12	0.01	0.16	0.02	0.34^*^	0.06
	SEN	0.49	0.07	0.99^*^	0.15	2.72^**^	0.44
TNFalpha	NS	0.22	0.09	0.62	0.11	1.59	0.19
	SEN	1.75	0.20	3.79^**^	0.35	7.00^**^	1.30

*<0.05, **<0.01, ***<0.001

IL-8 unit is µg/ml, and other cytokines units are pg/ml

**Table 2:** Cytokine levels in non-senescent and replicatively senescent cells.

		Water		HNG		MOTS-c	
		Mean	SE	Mean	SE	Mean	SE
IL-1beta	NS	0.42	0.03	0.46^**^	0.03	0.46^*^	0.02
	RS	0.60	0.04	0.68^**^	0.04	0.67^**^	0.04
IL-8	NS	496.5	17.40	552.4	16.03	568.5	3.38
	RS	798.4	4.14	989.3	8.78	969.0^***^	6.45
IL-10	NS	1.16	0.09	1.10	0.19	1.42	0.13
	RS	1.30	0.13	1.89	0.25	1.45	0.27
TNFalpha	NS	4.55	0.34	3.73^***^	0.22	3.94	0.52
	RS	6.26	0.47	7.01^***^	0.50	6.46	0.44

*<0.05, **<0.01, ***<0.001

All cytokines units are pg/ml.

**Table 3:** Cytokine levels in non-senescent and doxorubicin-senescent cells in response to JAK inhibitors.

		Water			JAKI			HNG			HNG+JAKI	
		Mean	SE		Mean		SE	Mean		SE	Mean	SE
IL-6	NS	2.70	0.15		1.96		0.08	1.96		0.17	2.78	0.35
	SEN	10.27	0.56		9.19		0.65	12.61*		0.27	9.19	0.65
IL-1 beta	NS	1.57	0.09		1.58		0.09	1.59		0.23	1.95	0.15
	SEN	6.94	0.57		8.15		0.26	6.83		0.15	8.838*	0.76
IL-8	NS	1.61	0.03		1.249***		0.04	1.63		0.08	1.78	0.01
	SEN	2.27	0.03		2.42		0.03	2.34		0.03	2.42	0.02
IL-10	NS	0.56	0.06		0.81		0.14	0.76		0.18	0.63	0.05
	SEN	2.31	0.10		3.88***		0.32	3.376**		0.27	3.356**	0.08
TNFalpha	NS	2.29	0.14		2.97		0.30	3.304		0.31	2.818	0.23
	SEN	8.90	0.10		13.11***		0.46	10.77*		0.56	12.57***	0.65

*<0.05, **<0.01, ***<0.001

IL-8 unit is µg/ml, and other cytokines units are pg/ml.

**Table 4:** Cytokine levels in non-senescent, replicatively senescent, and doxorubicin-senescent cells.

		Water		HNG		MOTS-c	
		Mean	SE	Mean	SE	Mean	SE
Eotaxin-3	NS	1.43	0.66	2.46	0.43	2.04	0.43
	RS	1.34	0.33	2.17	0.53	1.16	0.29
	SEN	1.10	0.95	2.66	0.97	1.64	0.28

All cytokines units are pg/ml.

## DISCUSSION

In this study, we examined key features of mitochondria physiology, including the levels of MDPs, primarily in doxorubicin-induced senescence. The senescence response to doxorubicin increased the number of mitochondria and the levels of mitochondrial respiration, mitochondrial-encoded proteins and the MDPs humanin and MOTS-c. mtDNA methylation is one potential mechanism underlying these mitochondrial changes. Methylation levels in the region of MT-CO1 declined, and COX1 expression increased. Targeting metabolic pathways in senescent cells might be a novel strategy to eliminate the senescent cells and modulate the SASP. As we previously showed, humanin and MOTS-c influence cellular energetics, so we asked whether humanin and MOTS-c were senolytic. Neither MDP was senolytic, despite increasing mitochondrial respiration and selected SASP factors in doxorubicin-induced and replicative senescence. These results support the hypothesis that bioenergetics in senescent cells play an important role in expression of certain SASP factors.

Recent studies show that mitochondrial depletion abolishes the development of certain senescent phenotypes, including the pro-inflammatory SASP [50]. The DNA damage response activates mTORC1, which promotes PGC-1β-dependent mitochondrial biogenesis and senescence phenotypes [50]. This study showed that mitochondria are required for timely cell cycle arrest and SASP production. We observed that mitochondrial DNA content increased 3-fold in doxorubicin-induced senescence. In addition, cellular ATP levels increased in senescent cells, which showed higher mitochondrial respiration, but not increased glycolysis. Mitochondria mass increases were demonstrated by greater mitochondrial DNA content and by increased mitochondrial protein in senescent cells.

It was recently shown that a JAK inhibitor decreases certain SASP factors, and systemic and adipose tissue inflammation in old mice [37]. Humanin binds to gp130 receptor and activates JAK/STAT pathways to increase cellular proliferation and viability [15]. As JAK activation correlates with certain SASP factors, and gp130/JAK/STAT pathway regulates IL-6 signaling [56,57], humanin treatment may increase SASP production in senescent cells through JAK activation. Here, we showed that IL-6 levels increased by humanin were diminished by JAK inhibitor, suggesting that humanin increases IL-6 in senescent cells via gp130/JAK pathway. However, we examined that IL-10 and TNF-α levels are still elevated by JAK inhibitor with humanin treatment. It suggests that other signaling pathways may be involved in humanin mediated SASP production.

As MOTS-c significantly lowered mitochondrial respiration in HEK293 cells, MOTS-c was expected to be a potent suppressor of mitochondrial respiration and SASP factor production in senescent cells [8]. Unexpectedly, MOTS-c increased mitochondrial respiration in senescent cells via increased fatty acid oxidation. Because senescent cells rely on fatty acids as a mitochondrial respiration fuel, the MOTS-c-mediated increase of fatty acid oxidation may increase energy production to support SASP production. Although senescence is identified by morphological changes and a characteristic gene expression signature, the precise characteristics and functions of senescent cells depend on cell type and context. The role of MOTS-c in energetics in senescent cells might differ depending on cell type and context. Further studies are required to understand the different MOTS-c roles in cellular energetics in cell types other than fibroblasts.

The elevated levels of humanin and MOTS-c in doxorubicin-induced senescence, and our finding that treatment with humanin and MOTS-c increased certain SASP factors, raise the question of whether the elevated levels of humanin and MOTS-c alone can induce senescence. We treated fibroblasts cells with humanin and MOTS-c for 10 days and examined SA-β-gal activity but found no SA-β-gal positive cells (*data not shown*). These results suggest that while enhanced energetics by humanin and MOTS-c does not cause senescence, these MDPs might help senescent cells maintain a senescent status and the production of SASP factors.

There are three possible strategies for the development of senolytic therapeutics: 1) diminish the induction of senescence by inhibiting p16 and p53/p21 activation, 2) eliminate the senescent cells by increasing apoptosis, 3) reduce the SASP. Because senescence has beneficial and detrimental effects depending upon the context, the third approach by reducing detrimental SASP production may be the most promising. Thus, targeting metabolism in senescence cells may prove to be an important strategy to reduce SASP production.

In summary, humanin and MOTS-c levels increased in senescent cells and exposure of senescent cells to these peptides increases mitochondrial respiration and certain SASP factors. These results suggest a therapeutic role for MDPs in mitochondrial energetics and SASP production in targeting senescent cells.

## METHODS

### Reagents and antibodies

HNG (a potent analogue of humanin with a glycine substitution, S14G) and MOTS-c were synthesized by Genscript (Piscataway, NJ, USA). HNG and MOTS-c were initially dissolved in Milli-Q water. Doxorubicin (Sigma, St. Louis, MO, USA) and hydrogen peroxide (Sigma) were used for inducing senescence status in culture. JAK inhibitor I (Millipore Sigma, Burlington, Massachusetts, USA) were used to inhibit JAK pathway. The following antibodies were used in this study: anti-GAPDH antibody (Cat. #5174S), anti-Lamin B1 antibody (Cat. #12586), Cleaved Caspase-3 (Asp175) antibody (Cat. #9661S), CPT1A antibody (Cat. #12252S), anti-rabbit IgG, HRP-linked antibody (Cat. #7074), and anti-mouse IgG, HRP-linked antibody (Cat. #7076). These antibodies are supplied by Cell Signaling Technology (Danvers, MA, USA). Anti-Tom20 antibody (Cat. #SC-17764; Santa Cruz Biotechnology, Dallas, Texas, USA), anti-glutaminase antibody (GLS; Cat. #701965; ThermoFisher Scientific, Wilmington, DE, USA), and anti-β-actin antibody (Cat. A5316; Sigma) were also used.

### Cell culture and treatment

Primary dermal fibroblasts from normal human adults (HDFa) were purchased from ATCC (Cat#. PCS-201-012; Manassas, VA, USA) and cultured in Fibroblast Basal Medium supplemented with Fibroblast Growth Kit-Low serum (ATCC) at 37° C in 5% CO_2_. HCA2 primary human fibroblasts from neonatal foreskin, from O. Pereira-Smith were cultured in in high glucose Dulbecco’s modified Eagle’s medium (DMEM; Life Technologies, Waltham, MA, USA) supplemented with 10% fetal bovine serum (FBS; Omega Scientific, Tarzana, CA, USA) at 37° C in 5% CO_2_ and 5% O_2_. For replicative senescence, primary dermal fibroblast cells were passaged every 3 days until they reach 20-25 population doublings (PDs) and were compared to cells at 5-6 PDs. Primary dermal fibroblasts were also induced to senesce by treating with doxorubicin or hydrogen peroxide [58,59]. Briefly, cells were treated with 250 nM doxorubicin for 24 h; the medium was replaced with complete medium, and then again, every 3 days for 10-14 days. Cells were treated with 200 μM hydrogen peroxide for 2 h; the medium was replaced with complete medium, and then cells were re-treated with hydrogen peroxide every 3 days for 7-10 days. To obtain the quiescent status of non-senescent cells, cells were switched to Fibroblast Basal Medium supplemented with Fibroblast Growth Kit- serum-free (ATCC) for 24h. For peptide treatments in serum free media, cells were switched to Fibroblast Basal Medium supplemented with Fibroblast Growth Kit- serum-free (ATCC) for 2 h followed by addition of the peptide for 1-3 days.

### Western blot analysis

Cells were lysed with RIPA Lysis and Extraction Buffer (ThermoFisher Scientific) plus the Halt protease & phosphatase inhibitor cocktail (ThermoFisher Scientific). The lysates were incubated on ice for 10 min, then disrupted using a sonicator, and the supernatants collected by centrifugation at 15,000 x g for 15 min at 4° C. The Pierce™ BCA Protein Assay Kit (ThermoFisher Scientific) was used to quantify protein content in the lysates. Proteins (10-40 μg) were separated on NuPAGE™ 4-12% Bis-Tris Protein Gels and blotted onto PVDF membranes (ThermoFisher Scientific). Membranes were incubated with primary antibody at 4° C overnight according to the manufacturer’s instructions. After several washes with Tris-buffered saline containing 0.1% Tween-20, membranes were incubated with the appropriate HRP-conjugated secondary antibody at room temperature (RT) for 1 h. Enhanced chemiluminescence was used to detect specific bands. Membranes were imaged on a Bio-Rad ChemiDoc XRS^+^ imager. Relative intensities of the bands were measured using Image J, a free program provided by National Institute of Health (Bethesda, Maryland, USA).

### Immunocytochemistry

Primary dermal fibroblasts plated on coverslips were fixed with 4% paraformaldehyde for 10 min at RT. After fixation, cells were permeabilized with 0.2% Triton X-100 in phosphate-buffered saline (PBS) for 10 min at RT and blocked in PBS containing 0.2% Triton X-100 and 1% bovine serum albumin (BSA) for 1-h at RT. Cells were then incubated with mouse anti-Tom20 antibody (1:100; Santa Cruz Biotechnology) in PBS containing 0.2% Triton X-100 and 1% BSA at 4° C overnight. After three washes with PBS, cells were incubated with Alexa Fluor 488-conjugated donkey anti-mouse IgG (1:200; Invitrogen) and Alexa Fluor 568-conjugated donkey anti-rabbit IgG (1:200; Invitrogen) in PBS containing 0.2% Triton X-100 and 1% BSA for 1-h at RT in the dark. Nuclei were stained for 5 min at RT in PBS containing Hoechst 33258 (2mg/ml; Invitrogen). Coverslips were mounted with ProLong Gold antifade reagent (Invitrogen) and observed under an LSM780 confocal microscope (Carl Zeiss, Germany).

### DNA extraction and mitochondrial DNA copy number measurement

Genomic DNA from 300,000 cells was extracted with DNeasy Blood & Tissue Kits (Qiagen, Valencia, CA, USA). The mitochondrial copy number was estimated by real-time PCR (CFX Connect Real-Time System, Biorad) using two mtDNA targets (ND1, CYB) and two nuclear DNA targets (β-actin, 36B4) (IDT, CA, USA). Real-time PCR was performed by using SsoAdvancedTM Universal SYBR® Green Supermix, following the protocol provided by the manufacturer. The ratio of mtDNA to nuclear DNA was calculated by averaging the differences in amplification efficiency between ND1 and β-actin (∆Ct) and between CYB and 36B4 (∆Ct). The primers used for amplification are: ND1 Forward primer (5’gaagtcaccctagccatcattc3’) and Reverse primer (5’gcaggagtaatcagaggtgttc3’); CYB Forward primer (5’ ctaggcgacccagacaattatac3’) and Reverse primer (5’ ttagggacggatcggagaat3’); β actin Forward primer (5’ actcttccagccttccttcc3’) and Reverse primer (5’ ggcaggacttagcttccaca3’); 36B4 Forward primer (5’ ggaatgtgggctttgtgttc3’) and Reverse primer (5’ cccaattgtccccttacctt3’).

### Cellular ATP measurement

Intracellular ATP levels were determined using an ATP Colorimetric/Fluorometric Assay Kit (BioVision, Inc., Milpitas, CA, USA) and carried out according to the manufacturer’s protocol. Briefly, 1x 10^6^ cells were lysed in ATP Assay Buffer, then homogenized using Amicon 10kDa Ultra-0.5 mL Centrifugal Filters (Millipore Sigma, Billerica, MA, USA). Lysates were transferred to a 96- well plate, and incubated with Reaction Buffer for 30 min at RT. Absorbance from standard curve and samples was measured on a plate spectrophotometer (Molecular Designs, Sunnyvale, CA) at 579 nm.

### Glucose uptake assay

Cellular glucose uptake was determined using a Glucose Uptake Cell-Based Assay Kit (Cayman chemical, Ann Arbor, MI, USA) according to the manufacturer’s protocol. The kit uses 2-NBDG, a fluorescently-labeled deoxyglucose analog, as a probe for detecting glucose taken up by cells. 2-NBDG was measured on a plate spectrophotometer (Molecular Designs) with fluorescent filters (excitation/emission = 485/535 nm).

### Measurement of mitochondrial energetics (OCR, ECAR, Mito Fuel Usage)

Cellular bioenergetics were determined by measuring the oxygen consumption rate (OCR) and extracellular acidification rate (ECAR) of the cells with the XF-96 Flux Analyzer (Seahorse Biosciences, North Billerica, MA, USA) at the Gerontology Seahorse Core Facility, USC. Seahorse XF Cell Mito Stress Test, Glycolysis Stress Test, and Mito Fuel Flex Test kits were used to examine OCR, ECAR, and mitochondrial fuel usage, respectively, according to the manufacturer’s instructions. A seeding density of 20,000 cells per well was used. OCR, basal respiration, mitochondrial respiration associated with cellular ATP production, and spare respiration capacity were estimated by challenging cells with oligomycin, FCCP (carbonyl cyanide 4-[trifluoromethoxy]phenylhydrazone), rotenone, and antimycin A. Briefly, mitochondrial ATP production is correlated with a decrease in OCR following exposure to oligomycin and spare respiratory capacity was estimated from the difference between FCCP-stimulated OCR and basal OCR. Regarding ECAR, glycolysis, glycolytic capacity and glycolytic reserve were estimated by challenging cells with glucose, oligomycin, and 2-deoxy-glucose (2-DG). Briefly, glycolysis was determined by glucose-induced response, glycolytic capacity was determined by the shift following oligomycin treatment, and glycolytic reserve was defined by the difference between glycolytic capacity and glycolysis rate. Mitochondrial fuel usage was determined by the rate of oxidation of each fuel (glucose, glutamine, and long-chain fatty acid) by measuring OCR of cells in the presence or absence of fuel pathway inhibitors. UK5099, BPTES, and Etomoxir were used as inhibitors of glucose, glutamine, and long-chain fatty acids oxidation pathways, respectively. All readings were normalized to total DNA content.

### Annexin V and PI staining

Muse Annexin V and Dead Cell Assay kit (Millipore Sigma) were used to stain cells for quantifying the live, early and late apoptosis, and cell death [60]. All procedures are done according to the manufacturer’s instructions. The Muse Cell Analyzer are used to quantify 5,000 cells.

### MTT assay

The viability of cells after humanin treatment were determined using the tetrazolium dye MTT [3-(4,5-dimethylthiazol-2-yl)-2,5-diphenyltetrazolium bromide] assay. All the treatments were done using 5,000 cells in 96 well plate. The purple insoluble formazan was read on a microplate reader at a wavelength of 570nm.

### Measurement of mtDNA methylation

mtDNA methylation was measured as described [54]. Briefly, mtDNA-containing cellular DNAs were treated with sodium bisulfite using an EZ DNA Methylation Kit (Zymo, CA), following the protocol provided by the manufacturer. The bisulfite-treated DNA was amplified by PCR in a 6 𝜇l reaction mixture containing 2 𝜇l 3× Klen-TaqI mix, 2 𝜇l template DNA, and 2 𝜇l 2.5 𝜇M primers. After initial denaturing at 95∘ C for 5 min, the DNA was amplified for 38 cycles at 95∘ C for 20 s, 62∘ C for 20 s of annealing, and 72∘ C for 20 s of extension and finally with extension at 72∘ C for 2 min. The PCR primers used to amplify the bisulfite-treated mtDNA are: CpG1 forward primer (5’ACGGAATAAGTTATTTTAGGGATAAT3’) and reverse primer (5’ACGAACCTTTAATAACGACTACACCA3’); CpG2 forward primer (5’CGAATTAAACCAAAAAATTAATTAATAC3’) and reverse primer (5’AGGAGTTTAAATTTTTTTATTTTTAGGAT3’); CpG4 forward primer (5’AAACCTACAAATAATAAAATATTTCATA3’) and reverse primer (5’TTTATTTTTAGGTTATATTTTAGATTAA3’).

The status of mtDNA methylation was determined by restriction enzymes that distinguish methylated and unmethylated DNAs. Specifically, DNAs were digested with Taq I at 65∘ C for 2h or HpyCH4IV at 37∘ C for 2h and separated on 3% agarose gel. Taq I recognizes methylated TCGA sites and HpyCH4IV digests methylated ACGT sites. After treatment with sodium bisulfate, unmethylated cytosines were converted to uracils and were converted to TTGA and ATGT after PCR, which are not digested by Taq I and HpyCH4IV, respectively. The methylated and unmethylated bands were scanned for quantitation [54].

### MDP measurements

The levels of MDPs including humanin, the small humanin-like peptides (SHLPs) and MOTS-c in conditioned medium were measured by an in-house sandwich ELISA as described [7,8,61]. Conditioned medium was generated by adding fibroblast Basal Medium supplemented with Fibroblast Growth Kit-Low serum (ATCC) for 24 h to cells. 100 μl of conditioned medium was used for the assay.

### SASP measurements

Conditioned medium was generated as described above. IL-6, IL-1β, IL-8, IL-10, and TNFα, Eotaxin, Eotaxin-3, IFN-γ, and I-TAC levels in the conditioned medium were determined using the customized V-PLEX pro-inflammatory panel 1 (MESO SCALE DISCOVERY, Rockville, MD, USA) according to the manufacturer’s protocols. For normalization, the concentrations obtained with this assay were divided by the number of cells in each sample.

### Statistical analysis

Data are presented as mean ± S.E.M. Significant differences were determined by Student’s *t*-tests, one-way ANOVA followed by Tukey’s *post hoc* test using GraphPad Prism 5 software. Values of *<0.05, **<0.01, ***<0.001 were considered statistically significant.

## Supplementary Material

Supplementary File

## References

[1] Liu B, Du Q, Chen L, Fu G, Li S, Fu L, Zhang X, Ma C, Bin C. CpG methylation patterns of human mitochondrial DNA. Sci Rep. 2016; 6:23421. 10.1038/srep23421 26996456 PMC4800444

[2] Iacobazzi V, Castegna A, Infantino V, Andria G. Mitochondrial DNA methylation as a next-generation biomarker and diagnostic tool. Mol Genet Metab. 2013; 110:25–34. 10.1016/j.ymgme.2013.07.012 23920043

[3] Shock LS, Thakkar PV, Peterson EJ, Moran RG, Taylor SM. DNA methyltransferase 1, cytosine methylation, and cytosine hydroxymethylation in mammalian mitochondria. Proc Natl Acad Sci USA. 2011; 108:3630–35. 10.1073/pnas.1012311108 21321201 PMC3048134

[4] Saini SK, Mangalhara KC, Prakasam G, Bamezai RN. DNA Methyltransferase1 (DNMT1) Isoform3 methylates mitochondrial genome and modulates its biology. Sci Rep. 2017; 7:1525. 10.1038/s41598-017-01743-y 28484249 PMC5431478

[5] Mawlood SK, Dennany L, Watson N, Dempster J, Pickard BS. Quantification of global mitochondrial DNA methylation levels and inverse correlation with age at two CpG sites. Aging (Albany NY). 2016; 8:636–41. 10.18632/aging.100892 26887692 PMC4925819

[6] Ikonen M, Liu B, Hashimoto Y, Ma L, Lee KW, Niikura T, Nishimoto I, Cohen P. Interaction between the Alzheimer’s survival peptide humanin and insulin-like growth factor-binding protein 3 regulates cell survival and apoptosis. Proc Natl Acad Sci USA. 2003; 100:13042–47. 10.1073/pnas.2135111100 14561895 PMC240741

[7] Cobb LJ, Lee C, Xiao J, Yen K, Wong RG, Nakamura HK, Mehta HH, Gao Q, Ashur C, Huffman DM, Wan J, Muzumdar R, Barzilai N, Cohen P. Naturally occurring mitochondrial-derived peptides are age-dependent regulators of apoptosis, insulin sensitivity, and inflammatory markers. Aging (Albany NY). 2016; 8:796–809. 10.18632/aging.100943 27070352 PMC4925829

[8] Lee C, Zeng J, Drew BG, Sallam T, Martin-Montalvo A, Wan J, Kim SJ, Mehta H, Hevener AL, de Cabo R, Cohen P. The mitochondrial-derived peptide MOTS-c promotes metabolic homeostasis and reduces obesity and insulin resistance. Cell Metab. 2015; 21:443–54. 10.1016/j.cmet.2015.02.009 25738459 PMC4350682

[9] Muzumdar RH, Huffman DM, Atzmon G, Buettner C, Cobb LJ, Fishman S, Budagov T, Cui L, Einstein FH, Poduval A, Hwang D, Barzilai N, Cohen P. Humanin: a novel central regulator of peripheral insulin action. PLoS One. 2009; 4:e6334. 10.1371/journal.pone.0006334 19623253 PMC2709436

[10] Gong Z, Tas E, Muzumdar R. Humanin and age-related diseases: a new link? Front Endocrinol (Lausanne). 2014; 5:210. 10.3389/fendo.2014.00210 25538685 PMC4255622

[11] Bachar AR, Scheffer L, Schroeder AS, Nakamura HK, Cobb LJ, Oh YK, Lerman LO, Pagano RE, Cohen P, Lerman A. Humanin is expressed in human vascular walls and has a cytoprotective effect against oxidized LDL-induced oxidative stress. Cardiovasc Res. 2010; 88:360–66. 10.1093/cvr/cvq191 20562421 PMC2952532

[12] Oh YK, Bachar AR, Zacharias DG, Kim SG, Wan J, Cobb LJ, Lerman LO, Cohen P, Lerman A. Humanin preserves endothelial function and prevents atherosclerotic plaque progression in hypercholesterolemic ApoE deficient mice. Atherosclerosis. 2011; 219:65–73. 10.1016/j.atherosclerosis.2011.06.038 21763658 PMC3885346

[13] Niikura T, Sidahmed E, Hirata-Fukae C, Aisen PS, Matsuoka Y. A humanin derivative reduces amyloid beta accumulation and ameliorates memory deficit in triple transgenic mice. PLoS One. 2011; 6:e16259. 10.1371/journal.pone.0016259 21264226 PMC3022031

[14] Hashimoto Y, Niikura T, Tajima H, Yasukawa T, Sudo H, Ito Y, Kita Y, Kawasumi M, Kouyama K, Doyu M, Sobue G, Koide T, Tsuji S, et al. A rescue factor abolishing neuronal cell death by a wide spectrum of familial Alzheimer’s disease genes and Abeta. Proc Natl Acad Sci USA. 2001; 98:6336–41. 10.1073/pnas.101133498 11371646 PMC33469

[15] Kim SJ, Guerrero N, Wassef G, Xiao J, Mehta HH, Cohen P, Yen K. The mitochondrial-derived peptide humanin activates the ERK1/2, AKT, and STAT3 signaling pathways and has age-dependent signaling differences in the hippocampus. Oncotarget. 2016; 7:46899–912. 10.18632/oncotarget.10380 27384491 PMC5216912

[16] Hashimoto Y, Suzuki H, Aiso S, Niikura T, Nishimoto I, Matsuoka M. Involvement of tyrosine kinases and STAT3 in Humanin-mediated neuroprotection. Life Sci. 2005; 77:3092–104. 10.1016/j.lfs.2005.03.031 16005025

[17] Hashimoto Y, Kurita M, Aiso S, Nishimoto I, Matsuoka M. Humanin inhibits neuronal cell death by interacting with a cytokine receptor complex or complexes involving CNTF receptor alpha/WSX-1/gp130. Mol Biol Cell. 2009; 20:2864–73. 10.1091/mbc.e09-02-0168 19386761 PMC2695794

[18] Guo B, Zhai D, Cabezas E, Welsh K, Nouraini S, Satterthwait AC, Reed JC. Humanin peptide suppresses apoptosis by interfering with Bax activation. Nature. 2003; 423:456–61. 10.1038/nature01627 12732850

[19] Klein LE, Cui L, Gong Z, Su K, Muzumdar R. A humanin analog decreases oxidative stress and preserves mitochondrial integrity in cardiac myoblasts. Biochem Biophys Res Commun. 2013; 440:197–203. 10.1016/j.bbrc.2013.08.055 23985350 PMC3853355

[20] Sreekumar PG, Ishikawa K, Spee C, Mehta HH, Wan J, Yen K, Cohen P, Kannan R, Hinton DR. The Mitochondrial-Derived Peptide Humanin Protects RPE Cells From Oxidative Stress, Senescence, and Mitochondrial Dysfunction. Invest Ophthalmol Vis Sci. 2016; 57:1238–53. 10.1167/iovs.15-17053 26990160 PMC4811181

[21] Zhao ST, Zhao L, Li JH. Neuroprotective Peptide humanin inhibits inflammatory response in astrocytes induced by lipopolysaccharide. Neurochem Res. 2013; 38:581–88. 10.1007/s11064-012-0951-6 23277413

[22] Zapała B, Kaczyński Ł, Kieć-Wilk B, Staszel T, Knapp A, Thoresen GH, Wybrańska I, Dembińska-Kieć A. Humanins, the neuroprotective and cytoprotective peptides with antiapoptotic and anti-inflammatory properties. Pharmacol Rep. 2010; 62:767–77. 10.1016/S1734-1140(10)70337-6 21098860

[23] Ming W, Lu G, Xin S, Huanyu L, Yinghao J, Xiaoying L, Chengming X, Banjun R, Li W, Zifan L. Mitochondria related peptide MOTS-c suppresses ovariectomy-induced bone loss via AMPK activation. Biochem Biophys Res Commun. 2016; 476:412–19. 10.1016/j.bbrc.2016.05.135 27237975

[24] Hodes RJ, Sierra F, Austad SN, Epel E, Neigh GN, Erlandson KM, Schafer MJ, LeBrasseur NK, Wiley C, Campisi J, Sehl ME, Scalia R, Eguchi S, et al. Disease drivers of aging. Ann N Y Acad Sci. 2016; 1386:45–68. 10.1111/nyas.13299 27943360 PMC5373660

[25] Demaria M, Ohtani N, Youssef SA, Rodier F, Toussaint W, Mitchell JR, Laberge RM, Vijg J, Van Steeg H, Dollé ME, Hoeijmakers JH, de Bruin A, Hara E, Campisi J. An essential role for senescent cells in optimal wound healing through secretion of PDGF-AA. Dev Cell. 2014; 31:722–33. 10.1016/j.devcel.2014.11.012 25499914 PMC4349629

[26] Hartley AV, Martin M, Lu T. Aging: Cancer - an unlikely couple. Aging (Albany NY). 2017; 9:1949–50. 10.18632/aging.101295 28952453 PMC5636664

[27] Burd CE, Sorrentino JA, Clark KS, Darr DB, Krishnamurthy J, Deal AM, Bardeesy N, Castrillon DH, Beach DH, Sharpless NE. Monitoring tumorigenesis and senescence in vivo with a p16(INK4a)-luciferase model. Cell. 2013; 152:340–51. 10.1016/j.cell.2012.12.010 23332765 PMC3718011

[28] Kuilman T, Peeper DS. Senescence-messaging secretome: SMS-ing cellular stress. Nat Rev Cancer. 2009; 9:81–94. 10.1038/nrc2560 19132009

[29] Özcan S, Alessio N, Acar MB, Mert E, Omerli F, Peluso G, Galderisi U. Unbiased analysis of senescence associated secretory phenotype (SASP) to identify common components following different genotoxic stresses. Aging (Albany NY). 2016; 8:1316–29. 10.18632/aging.100971 27288264 PMC4993333

[30] Kang TW, Yevsa T, Woller N, Hoenicke L, Wuestefeld T, Dauch D, Hohmeyer A, Gereke M, Rudalska R, Potapova A, Iken M, Vucur M, Weiss S, et al. Senescence surveillance of pre-malignant hepatocytes limits liver cancer development. Nature. 2011; 479:547–51. 10.1038/nature10599 22080947

[31] Freund A, Orjalo AV, Desprez PY, Campisi J. Inflammatory networks during cellular senescence: causes and consequences. Trends Mol Med. 2010; 16:238–46. 10.1016/j.molmed.2010.03.003 20444648 PMC2879478

[32] Fougère B, Boulanger E, Nourhashémi F, Guyonnet S, Cesari M. Chronic Inflammation: Accelerator of Biological Aging. J Gerontol A Biol Sci Med Sci. 2017; 72:1218–25. 10.1093/gerona/glw240 28003373

[33] Chang J, Wang Y, Shao L, Laberge RM, Demaria M, Campisi J, Janakiraman K, Sharpless NE, Ding S, Feng W, Luo Y, Wang X, Aykin-Burns N, et al. Clearance of senescent cells by ABT263 rejuvenates aged hematopoietic stem cells in mice. Nat Med. 2016; 22:78–83. 10.1038/nm.4010 26657143 PMC4762215

[34] Baker DJ, Wijshake T, Tchkonia T, LeBrasseur NK, Childs BG, van de Sluis B, Kirkland JL, van Deursen JM. Clearance of p16Ink4a-positive senescent cells delays ageing-associated disorders. Nature. 2011; 479:232–36. 10.1038/nature10600 22048312 PMC3468323

[35] Jeon OH, Kim C, Laberge RM, Demaria M, Rathod S, Vasserot AP, Chung JW, Kim DH, Poon Y, David N, Baker DJ, van Deursen JM, Campisi J, Elisseeff JH. Local clearance of senescent cells attenuates the development of post-traumatic osteoarthritis and creates a pro-regenerative environment. Nat Med. 2017; 23:775–81. 10.1038/nm.4324 28436958 PMC5785239

[36] Ogrodnik M, Miwa S, Tchkonia T, Tiniakos D, Wilson CL, Lahat A, Day CP, Burt A, Palmer A, Anstee QM, Grellscheid SN, Hoeijmakers JH, Barnhoorn S, et al. Cellular senescence drives age-dependent hepatic steatosis. Nat Commun. 2017; 8:15691. 10.1038/ncomms15691 28608850 PMC5474745

[37] Xu M, Palmer AK, Ding H, Weivoda MM, Pirtskhalava T, White TA, Sepe A, Johnson KO, Stout MB, Giorgadze N, Jensen MD, LeBrasseur NK, Tchkonia T, Kirkland JL. Targeting senescent cells enhances adipogenesis and metabolic function in old age. eLife. 2015; 4:e12997. 10.7554/eLife.12997 26687007 PMC4758946

[38] Zhu Y, Tchkonia T, Fuhrmann-Stroissnigg H, Dai HM, Ling YY, Stout MB, Pirtskhalava T, Giorgadze N, Johnson KO, Giles CB, Wren JD, Niedernhofer LJ, Robbins PD, Kirkland JL. Identification of a novel senolytic agent, navitoclax, targeting the Bcl-2 family of anti-apoptotic factors. Aging Cell. 2016; 15:428–35. 10.1111/acel.12445 26711051 PMC4854923

[39] Baar MP, Brandt RM, Putavet DA, Klein JD, Derks KW, Bourgeois BR, Stryeck S, Rijksen Y, van Willigenburg H, Feijtel DA, van der Pluijm I, Essers J, van Cappellen WA, et al. Targeted Apoptosis of Senescent Cells Restores Tissue Homeostasis in Response to Chemotoxicity and Aging. Cell. 2017; 169:132–147.e16. 10.1016/j.cell.2017.02.031 28340339 PMC5556182

[40] Zhu Y, Tchkonia T, Pirtskhalava T, Gower AC, Ding H, Giorgadze N, Palmer AK, Ikeno Y, Hubbard GB, Lenburg M, O’Hara SP, LaRusso NF, Miller JD, et al. The Achilles’ heel of senescent cells: from transcriptome to senolytic drugs. Aging Cell. 2015; 14:644–58. 10.1111/acel.12344 25754370 PMC4531078

[41] Wiley CD, Campisi J. From Ancient Pathways to Aging Cells-Connecting Metabolism and Cellular Senescence. Cell Metab. 2016; 23:1013–21. 10.1016/j.cmet.2016.05.010 27304503 PMC4911819

[42] Bittles AH, Harper N. Increased glycolysis in ageing cultured human diploid fibroblasts. Biosci Rep. 1984; 4:751–56. 10.1007/BF01128816 6509159

[43] Quijano C, Cao L, Fergusson MM, Romero H, Liu J, Gutkind S, Rovira II, Mohney RP, Karoly ED, Finkel T. Oncogene-induced senescence results in marked metabolic and bioenergetic alterations. Cell Cycle. 2012; 11:1383–92. 10.4161/cc.19800 22421146 PMC3350879

[44] Wiley CD, Velarde MC, Lecot P, Liu S, Sarnoski EA, Freund A, Shirakawa K, Lim HW, Davis SS, Ramanathan A, Gerencser AA, Verdin E, Campisi J. Mitochondrial Dysfunction Induces Senescence with a Distinct Secretory Phenotype. Cell Metab. 2016; 23:303–14. 10.1016/j.cmet.2015.11.011 26686024 PMC4749409

[45] Stolzing A, Coleman N, Scutt A. Glucose-induced replicative senescence in mesenchymal stem cells. Rejuvenation Res. 2006; 9:31–35. 10.1089/rej.2006.9.31 16608393

[46] Laberge RM, Sun Y, Orjalo AV, Patil CK, Freund A, Zhou L, Curran SC, Davalos AR, Wilson-Edell KA, Liu S, Limbad C, Demaria M, Li P, et al. MTOR regulates the pro-tumorigenic senescence-associated secretory phenotype by promoting IL1A translation. Nat Cell Biol. 2015; 17:1049–61. 10.1038/ncb3195 26147250 PMC4691706

[47] Gao C, Ning B, Sang C, Zhang Y. Rapamycin prevents the intervertebral disc degeneration via inhibiting differentiation and senescence of annulus fibrosus cells. Aging (Albany NY). 2018; 10:131–43. 10.18632/aging.101364 29348392 PMC5811247

[48] Pascal T, Debacq-Chainiaux F, Chrétien A, Bastin C, Dabée AF, Bertholet V, Remacle J, Toussaint O. Comparison of replicative senescence and stress-induced premature senescence combining differential display and low-density DNA arrays. FEBS Lett. 2005; 579:3651–59. 10.1016/j.febslet.2005.05.056 15963989

[49] Vander Heiden MG, Cantley LC, Thompson CB. Understanding the Warburg effect: the metabolic requirements of cell proliferation. Science. 2009; 324:1029–33. 10.1126/science.1160809 19460998 PMC2849637

[50] Correia-Melo C, Marques FD, Anderson R, Hewitt G, Hewitt R, Cole J, Carroll BM, Miwa S, Birch J, Merz A, Rushton MD, Charles M, Jurk D, et al. Mitochondria are required for pro-ageing features of the senescent phenotype. EMBO J. 2016; 35:724–42. 10.15252/embj.201592862 26848154 PMC4818766

[51] Strigun A, Wahrheit J, Niklas J, Heinzle E, Noor F. Doxorubicin increases oxidative metabolism in HL-1 cardiomyocytes as shown by 13C metabolic flux analysis. Toxicol Sci. 2012; 125:595–606. 10.1093/toxsci/kfr298 22048646

[52] Berthiaume JM, Wallace KB. Persistent alterations to the gene expression profile of the heart subsequent to chronic Doxorubicin treatment. Cardiovasc Toxicol. 2007; 7:178–91. 10.1007/s12012-007-0026-0 17901561

[53] Tokarska-Schlattner M, Lucchinetti E, Zaugg M, Kay L, Gratia S, Guzun R, Saks V, Schlattner U. Early effects of doxorubicin in perfused heart: transcriptional profiling reveals inhibition of cellular stress response genes. Am J Physiol Regul Integr Comp Physiol. 2010; 298:R1075–88. 10.1152/ajpregu.00360.2009 20053966

[54] Yu D, Du Z, Pian L, Li T, Wen X, Li W, Kim SJ, Xiao J, Cohen P, Cui J, Hoffman AR, Hu JF. Mitochondrial DNA Hypomethylation Is a Biomarker Associated with Induced Senescence in Human Fetal Heart Mesenchymal Stem Cells. Stem Cells Int. 2017; 2017:1764549–12. 10.1155/2017/1764549 28484495 PMC5397648

[55] Lee C, Kim KH, Cohen P. MOTS-c: A novel mitochondrial-derived peptide regulating muscle and fat metabolism. Free Radic Biol Med. 2016; 100:182–87. 10.1016/j.freeradbiomed.2016.05.015 27216708 PMC5116416

[56] Heinrich PC, Behrmann I, Müller-Newen G, Schaper F, Graeve L. Interleukin-6-type cytokine signalling through the gp130/Jak/STAT pathway. Biochem J. 1998; 334:297–314. 10.1042/bj3340297 9716487 PMC1219691

[57] Xu M, Tchkonia T, Ding H, Ogrodnik M, Lubbers ER, Pirtskhalava T, White TA, Johnson KO, Stout MB, Mezera V, Giorgadze N, Jensen MD, LeBrasseur NK, Kirkland JL. JAK inhibition alleviates the cellular senescence-associated secretory phenotype and frailty in old age. Proc Natl Acad Sci USA. 2015; 112:E6301–10. 10.1073/pnas.1515386112 26578790 PMC4655580

[58] Wang Z, Wei D, Xiao H. Methods of cellular senescence induction using oxidative stress. Methods Mol Biol. 2013; 1048:135–44. 10.1007/978-1-62703-556-9_11 23929103

[59] Demaria M, O’Leary MN, Chang J, Shao L, Liu S, Alimirah F, Koenig K, Le C, Mitin N, Deal AM, Alston S, Academia EC, Kilmarx S, et al. Cellular Senescence Promotes Adverse Effects of Chemotherapy and Cancer Relapse. Cancer Discov. 2017; 7:165–76. 10.1158/2159-8290.CD-16-0241 27979832 PMC5296251

[60] Khan A, Gillis K, Clor J, Tyagarajan K. Simplified evaluation of apoptosis using the Muse cell analyzer. Postepy Biochem. 2012; 58:492–96. 23662443

[61] Chin YP, Keni J, Wan J, Mehta H, Anene F, Jia Y, Lue YH, Swerdloff R, Cobb LJ, Wang C, Cohen P. Pharmacokinetics and tissue distribution of humanin and its analogues in male rodents. Endocrinology. 2013; 154:3739–44. 10.1210/en.2012-2004 23836030 PMC3776863

