# Supplementary material for: Mitochondrial peptides modulate mitochondrial function during cellular senescence
Source: Aging (Albany NY). 2018 Jun 10;10(6):1239–56. doi: 10.18632/aging.101463 (PMC6046248; doi:10.18632/aging.101463)
Supplement: Supplementary File [file aging-10-101463-s001.pdf]

SUPPLEMENTARY MATERIAL

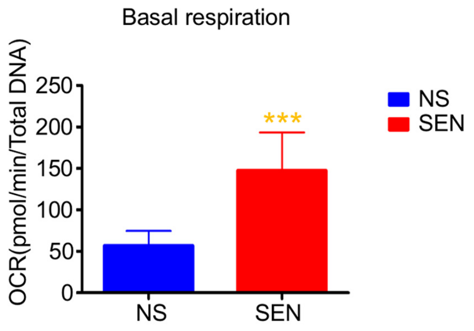

Supplementary Figure 1. Mitochondrial respiration was increased during hydrogen peroxide-induced senescence. Cellular oxygen consumption rate (OCR) in non-senescence and senescent cells. Data are reported as mean ± SEM of three independent experiments. Significant differences were determined with Student's *t*-tests. ***p<0.001. Abbreviations: NS, Non-senescent cells (quiescent); SEN, Senescent cells.

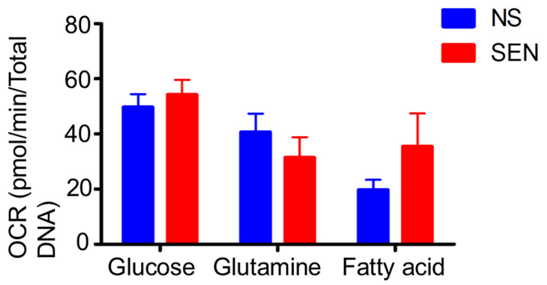

Supplementary Figure 2. Mitochondrial fuel usage during doxorubicin-induced senescence. Cellular oxygen consumption rate (OCR) in the presence of the inhibitors of the glucose, glutamine, and long chain fatty acid oxidation pathway in non-senescent and senescent cells (N=4).

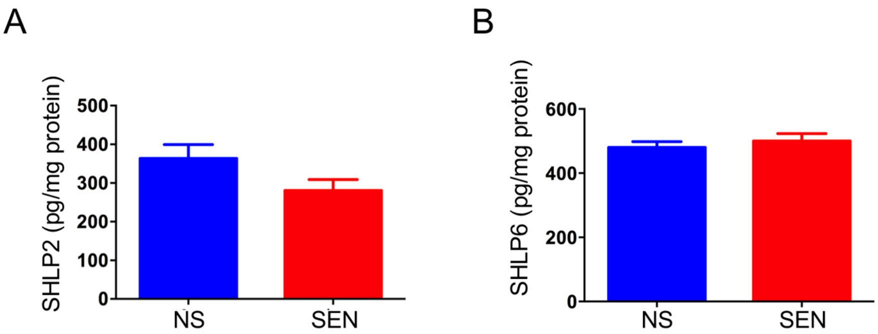

Supplementary Figure 3. SHLP2 and SHLP6 levels are not changed during doxorubicin-induced senescent cells. (**A**) SHLP2 and (**B**) SHLP6 levels were examined in doxorubicin-induced senescence (N=3).

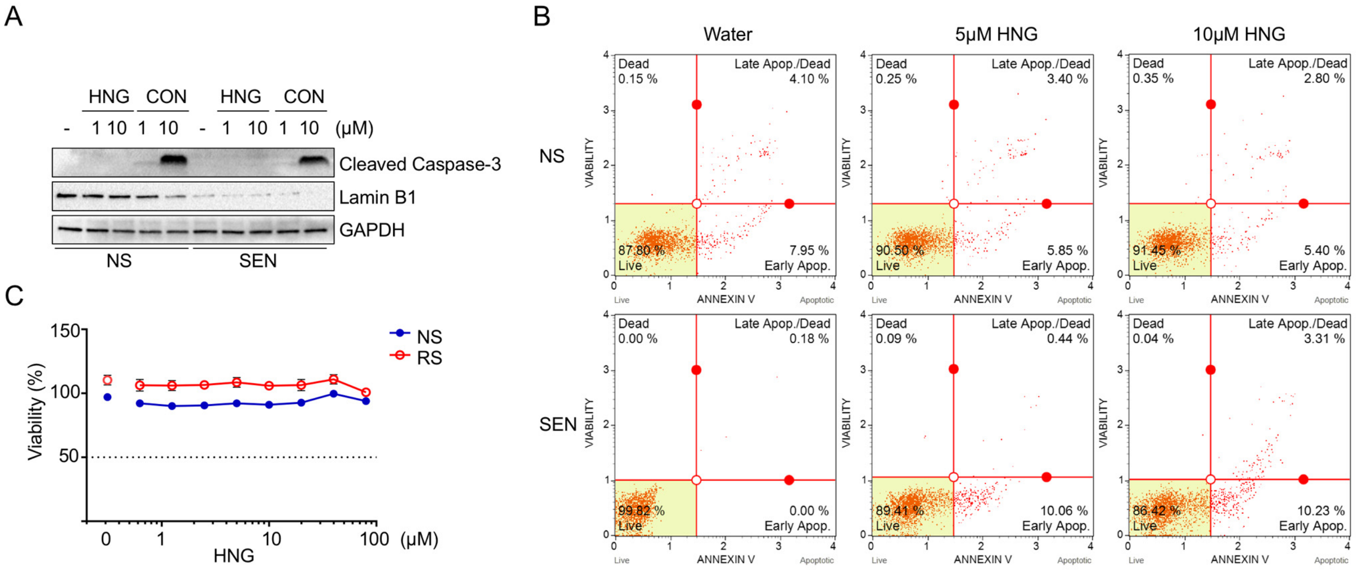

Supplementary Figure 4. Humanin analog, HNG, does not induce apoptosis. (**A**) Western blots of cleaved caspase-3. anti-Lamin B1 and anti-GAPDH antibody were used as a senescence marker and loading control, respectively. Control samples (CON) were used for positive control for cleaved caspase-3. (**B**) Flow cytometry of Annexin V and PI staining from HNG-treated non-senescent and doxorubicin-induced senescent cells (**C**) Viability of non-senescent and replicative senescent cells treated with HNG.
